# A survey of the knowledge, attitudes and practices on Zika virus in new York City

**DOI:** 10.1186/s12889-017-4991-3

**Published:** 2018-01-02

**Authors:** Gregory Samuel, Rianna DiBartolo-Cordovano, Insiya Taj, Audrey Merriam, Jean M. Lopez, Caroline Torres, Rafael A. Lantigua, Steven Morse, Bernard P. Chang, Cynthia Gyamfi-Bannerman, Kiran T. Thakur

**Affiliations:** 1 Department of Neurology, Columbia University Medical Center, 177 Fort Washington Avenue, New York, NY 10032 USA; 2 Department of Epidemiology, Columbia University Mailman School of Public Health, New York, NY USA; 3 Department of Obstetrics & Gynecology, Columbia University Medical Center, New York, NY USA; 4 Department of Internal Medicine, Columbia University Medical Center, New York, NY USA; 5 Department of Emergency Medicine, Columbia University Medical Center, New York, NY USA

**Keywords:** Zika virus, Health promotion, Maternal health

## Abstract

**Background:**

Over 900 travel-associated Zika virus cases have been identified in New York City (NYC), New York. A survey was administered in NYC adapted from the Knowledge, Attitudes, and Practices (KAP) survey on Zika virus developed by the World Health Organization (WHO).

**Methods:**

A standardized, self-administered, anonymous questionnaire was administered to a convenience sample in Manhattan and the Bronx from June 30th, 2016 to October 21st, 2016. Responses were grouped into six domains based on the content and structure of the questions and were summarized using descriptive statistics or converted into a continuous knowledge score and assessed for associations with pregnancy status and travel history using linear regression.

**Results:**

There were 224 respondents with a mean age of 33 (SD ± 11.6) with 77% (170/224) female and 24% (51/224) pregnant. The majority (98% (213/217)) were unable to identify all of the symptoms associated with acute Zika virus infection and all modes of transmission (97% (213/219)). Most participants (85% (187/219)) identified mosquitoes as a mode of transmission. 95% (116/122) reported an association between Zika virus and microcephaly. The most concerning aspect of Zika virus in 46% (91/200) was the risk of disabilities to babies, and risk of sexual transmission (25% (49/200)). When asked what precautions pregnant persons should to reduce the risk of transmission when traveling to a Zika endemic region, only 27% (50/185) identified using condoms during intercourse or refraining from intercourse while pregnant. Knowledge of Zika transmission is significantly positively associated with pregnancy status, but not with travel history.

**Conclusion:**

Our results indicate an overall poor understanding of Zika virus symptoms and possible complications, transmission modes, and current recommended prevention guidelines. Pregnancy is positively associated with *Knowledge of Zika Transmission*, but not other knowledge scores. Reported travel history to Zika endemic regions is not significantly associated with Zika knowledge. There is a need for implementing future public health interventions that particularly focus on protection against Zika transmission, that Zika is sexually transmitted, and risks that the Guillain-Barré Syndrome poses a risk to adults.

**Electronic supplementary material:**

The online version of this article (10.1186/s12889-017-4991-3) contains supplementary material, which is available to authorized users.

## Background

Zika virus is a single-stranded RNA virus of the *Flaviviridae* family, transmitted primarily through the bite of an infected *Aedes* species mosquito [1–3]. While approximately 80% of those infected do not develop symptoms, 20% of individuals develop a self-limited illness characterized by fever with maculopapular rash, arthralgia, or conjunctivitis [4]. More importantly, neurological manifestations can occur, as Zika infection has been identified as a trigger of Guillain-Barré Syndrome (GBS), an acute paralyzing condition [2]. Furthermore, a causal link has now been established between Zika virus infection during pregnancy and the risk of development of congenital anomalies [2].

Zika virus and its potential complications have been the focus of a significant global public health campaign. National and international public health organizations including the Centers for Disease Control (CDC), the Pan American Health Organization (PAHO), and the World Health Organization (WHO) have conducted regional and international public education campaigns on the risks of Zika virus infection, its symptoms, appropriate evaluation, and preventive behaviors in order to minimize transmission risk and complications [1, 4, 5]. Current understanding of Zika virus, its pathophysiology, transmission and complications have drawn international collaborations from epidemiologists, clinician scientists, and basic scientists.

The first large outbreak of Zika virus occurred on the island of Yap in 2007, when an estimated 73% of the population contracted the virus [6]. In 2015, a major outbreak of Zika virus occurred in Brazil, during which time there were marked increases in the numbers of infants born with congenital anomalies and GBS cases [6]. On February 1, 2016, the WHO declared the Zika virus outbreak a public health emergency of international concern given the rapid rise in microcephaly and other neurological syndromes seen in South America and the Pacific islands. Since 2007, 75 countries and territories have reported mosquito-borne Zika virus transmission [6]. There have been a total of 4431 laboratory confirmed cases of Zika virus reported in the United States between 2015 and 2016 [7]. The majority of these cases have been travel-associated, with locally transmitted Zika virus cases identified only in Florida and Texas [7]. New York City (NYC) has a largest number of Zika virus cases in the United States [2, 7]. Of the 1112 cases of definitive Zika virus infections identified in NYC, all have been associated with travel, with nearly half of those cases originating in the Dominican Republic [2, 8]. During summer 2016, the number of Zika virus test requests made to the NYC Department of Health and Mental Hygiene by and for women who had traveled to areas with active Zika virus transmission while pregnant increased weekly despite a travel warning being issued months before [9].

In response to the ongoing outbreak, the WHO released a survey kit of Knowledge, Attitudes and Practices (KAP) surrounding Zika Virus, in order to obtain information that could inform public health outreach efforts at a community level [10]. While a growing body of scientific literature has elucidated pathophysiological mechanisms and several clinical research studies have evaluated risks of severe neurological sequelae, little work has studied community knowledge on preventive practices and possible complications, particularly the at-risk populations of pregnant women and those frequently traveling to or living in Zika-endemic regions [11]. We hypothesize that pregnant persons and persons who had traveled to an area with active Zika virus transmission (the Caribbean, Central or South America) in the year prior to taking the survey, have greater knowledge of Zika transmission prevention guidelines and health complications than do non-pregnant persons or persons who did not travel to an epidemic area. In this study, we aim to describe the knowledge, attitudes, and practices surrounding Zika virus in particularly at-risk communities in NYC. An understanding of current knowledge and perceptions of Zika virus will allow government and non-government organizations to gauge the need for and impacts of public health awareness campaigns.

## Methods

### Study design, setting and sites

A standardized, self-administered, anonymous questionnaire based on the Knowledge, Attitudes and Practices Survey produced by the WHO was developed [10]. The survey kit from the WHO contained a total of 155 questions, divided into two question banks: ‘General ‘and ‘Sexual reproductive health’. Questions from the ‘Sexual reproductive health’ question bank appeared in the ‘General’ question bank. The ‘General’ question bank contained 112 questions total; 26 questions on knowledge, 34 on attitudes, 41 questions on practices, and 11 questions on participant demographic information. In order to facilitate completion within 30 min, our survey was shortened to 56 questions; with 20 questions on knowledge, 7 questions on attitudes, 10 questions on practices, 11 questions on participant demographics and 8 questions on participant Medical and Social history, including recent travel. An English version of the survey was translated and back-translated into Spanish. Prior to study initiation, seven questionnaires were administered to community members at a local health center in Northern Manhattan as a pilot test evaluating for timing, interpretation, and clarity of questions and instructions. Confusing and/or problematic questions were then modified or excluded prior to the initiation of the actual study.

We were particularly interested in understanding the knowledge, attitudes and practices of pregnant people and people from or frequently traveling to the Dominican Republic or other areas with active Zika transmission. The Northern Manhattan (north of 125th street) and Bronx areas of NYC contain the largest population of immigrants from the Dominican Republic, and from Latin America overall, in NYC. Thus, we administered our study specifically in the Northern Manhattan neighborhood of Inwood and Washington Heights and the University Heights neighborhood the Bronx, whose populations contain the largest immigrant group from the Dominican Republic. Immigrants from the Dominican Republic comprise 69%, 60%, and 59% of the populations of Inwood, Washington Heights, and University Heights respectively [12]. We distributed our questionnaire in several hospital and community settings including clinics, libraries, community colleges and health centers. To ensure the inclusion of pregnant persons in our study we also administered the survey to obstetric patients at Columbia University Medical Center facilities in Midtown and Washington Heights neighborhoods in Manhattan (NYC). After participants completed and returned questionnaires they were provided educational materials on Zika virus produced by the NYC Department of Mental Health and Hygiene, CDC and WHO.

### Data collection

A member of the research team visited each location between one and five times. Participants were recruited via convenience sampling and were approached by a member of the research team who explained the survey purpose and received verbal assent to participate. Participants were included if they were ≥16 years of age and were able to read and write in English or Spanish. They were provided a hard copy survey in the language (Spanish or English) of their preference. Surveys were self-administered, anonymous, and no personal identifying information was collected. Given the anonymous nature of our survey, written consent from participants was not obtained prior to participation. Although researchers did not determine the response rate, they estimate that approximately 1/3 of the persons approached to take the survey agreed to participate.

### Statistical analysis

Survey questions were organized into six domains based on the content/structure of the question and the information it was attempting to assess; attitudes towards Zika, practices related to Zika, general Zika knowledge, knowledge of Zika transmission, knowledge of Zika complications, and knowledge of Zika guidelines. More information about these domains as well as the questions included in each can be found in the Additional file 1. Given that questions in the first 3 domains were subjective, responses were analyzed using descriptive statistics to determine the percentage of participants selecting each survey response (m) of the total number of participants who answered the question (n). Questions in the last three domains had objectively correct answers, and therefore, were coded as continuous knowledge scores. Participants received points for selecting the correct answers to the questions that comprise the *Knowledge of Zika Transmission, Knowledge of Zika Guidelines* and *Knowledge of Zika Complications* domains. The score for the component questions that comprised these domains was calculated as the number of correct responses divided by the number of total possible points. A participant’s score for the continuous knowledge scores for the domains *Knowledge of Zika Transmission, Knowledge of Zika Guidelines* and *Knowledge of Zika Complications* was calculated as the average of the correct responses to all questions within the domain, and was multiplied by 100 for ease of interpretability. Knowledge scores range from 0 to 100. Participants that were missing responses for more than half of the questions within a domain were considered missing for that knowledge score. Linear regression was utilized to assess the association between *Knowledge of Zika Transmission* and *Complications* and participant characteristics. *Knowledge of Zika Guidelines* was not normally distributed and was not assessed using linear regression. Data was analyzed using SAS 9.4 (2002–2012, Statistical Analysis Software, Inc.). Of the 233 total completed surveys, seven pilot surveys and two surveys of individuals who only answered name and demographic information were excluded entirely from analysis.

One participant indicated both that they were pregnant and that their gender was male. We decided not to assume that this was a mistake because the survey question assessed gender, but only provided sexes as answer options. It is possible that a person was pregnant and selected the answer that best represented their gender, regardless of sex characteristics. This participant was excluded from regressions assessing the relationship between pregnancy and knowledge and from stratifications by pregnancy, because a single person does not provide enough statistical power to assess interaction between gender and pregnancy status and results in off-support data. We retained this participant in all other analyses and stratifications.

### Ethics

Our study was granted exemption by the Institutional Review Board (IRB) at Columbia University Medical Center (NYC, New York).

## Results

### Demographics

Most of the participants (77% (170/221)) were female and 24% (51/214) reported being pregnant at the time of survey completion. Mean age was 33 years (SD ± 11.62), with 42% (95/221) of the participants identifying English as their primary language and 43% (96/221) identifying Spanish as their primary language. Just over one-third (34% (77/224)) of the completed surveys were administered in the Bronx, and the remaining distributed throughout the aforementioned Manhattan locations. The majority of participants (54% (114/199)) reported living in the Bronx followed by Northern Manhattan (north of 125th street) (23% (50/199)). Most participants (64% (136/213)) reported being born outside of the United States; the most common nation of origin was the Dominican Republic (34% (72/213)). Nearly one-third (31% (69/224)) reported traveling to the Caribbean, Central America or South America within the last year, and among participants who have recently traveled, the most common destination was the Dominican Republic (69% (47/68)) followed by Mexico (12% (8/68)) [Table 1].

**Table 1:** Demographic characteristics of all survey participants

Total participants (*N* = 224)	m (%)
Age (*n* = 208)	
*mean (+/− sd)*	33 (+/− 11.6)
Gender (*n* = 221)	
Male	51 (23.1%)
Female	170 (76.9%)
Pregnant (*n* = 224)	51 (24.3%)
Trimester of Pregnancy (*n* = 48)	
First Trimester	14 (29.2%)
Second Trimester	18 (37.5%)
Third Trimester	16 (33.3%)
Primary Language (*n* = 224)	
English	95 (42.4%)
Spanish	96 (42.8%)
Other	33 (14.7%)
Where participants live (*n* = 216)	
Northern Manhattan (north of 125th street)	50 (23.2%)
Upper Manhattan (between 59th street and 125th street)	13 (6.0%)
Midtown Manhattan (between 23rd street and 59th street)	4 (1.9%)
Downtown Manhattan (south of 23rd street)	5 (2.3%)
Bronx	114 (52.8%)
Brooklyn	10 (4.6%)
Queens	3 (1.4%)
Staten Island	0 (0.0%)
Lives outside NYC	17 (7.9%)
Birthplace (*n* = 215)	
North America	81 (37.7%)
Central America	6 (2.8%)
South America	7 (3.3%)
Caribbean	79 (36.7%)
Europe	10 (4.7%)
Asia	7 (3.3%)
Africa	24 (11.2%)
Australia	1 (0.5%)
Traveled to the Caribbean, South America or Central America within the last 12 months (*n* = 224)	69 (30.8%)
^a^Travel Location (*n* = 68)	
Caribbean	62 (91.2%)
Dominican Republic	47 (69.1%)
Mexico	8 (11.8%)
South America	7 (10.3%)
Central America	5 (7.4%)
Other	2 (2.9%)
When last traveled (*n* = 70)	
Within the last week	3 (1.4%)
1–4 weeks ago	4 (1.8%)
1–3 months ago	20 (9.1%)
3–6 months ago	13 (5.9%)
6–12 months ago	30 (13.6%)

*n* = number of participants who responded to survey question; *m* = number who provided the response indicated; *n* = number of participants who responded to survey question; ^a^=Multiple responses were possible for this question; “Other” travel locations were Guinea and Spain

### Knowledge

The majority of participants (84% (187/222)) reported that they have heard of Zika virus before this survey. Most participants first heard of Zika through radio / TV /posters / newspapers (70% (132/190)), followed by internet/social media (27% (52/190)), and friends/family/neighbors (16% (30/190)). Only 9% (17/190) reported they heard about Zika virus from healthcare providers and only 7% (13/190) through government announcement. Nearly two-thirds of all the participants and half of the pregnant participants (50% (24/48)) believed they did not have enough information about Zika (64% (138/214). Most participants 84% (122/145)) indicated that they wanted more information [Table 2].

**Table 2:** General Zika knowledge, stratified by pregnancy status and gender

Total participants (*N* = 223)	Pregnant	Non-pregnant		
		All	Females	Males
	m (%)	m (%)	m (%)	m (%)
Had heard about Zika prior to taking the survey: (*n* = 222)	47 (94%)	128 (80.5%)	98 (84.4%)	28 (70.0%)
First head about Zika: (*n* = 189)				
Many years ago	0	9 (6.9%)	4 (4.0%)	4 (14.8%)
Last year	28 (59.8%)	62 (47.7%)	54 (53.5%)	8 (29.6%)
In the last few months	19 (40.4%)	57 (43.9%)	41 (40.6%)	15 (55.6%)
In the last week	0	2 (1.5%)	2 (20.%)	0
^a^First heard about Zika from: (*n* = 190)				
Family, Friends or neighbors	1 (2.1%)	28 (16.3%)	23 (16.4%)	5 (17.2%)
Church/ Community event	0	1 (0.6%)	1 (0.7%)	0
Healthcare workers/Private doctor/ Pharmacy	6 (12.5%)	10 (5.8%)	10 (7.1%)	0
Radio/ TV/ Posters/ Newspapers	34 (70.8%)	91 (52.9%)	72 (51.4%)	17 (58.6%)
Internet/ Social media	17 (35.4%)	32 (18.6%)	25 (17.9%)	6 (20.7%)
Government announcement	3 (6.25%)	10 (5.8%)	9 (6.4%)	1 (3.4%)
Think it is possible to get Zika in their community/local area now: (*n* = 211)	28 (57.1%)	88 (58.3%)	66 (58.9%)	22 (57.9%)
Had heard of Microcephaly before: (*n* = 218)	35 (71.4%)	90 (57.7%)	69 (60.0%)	19 (50.0%)
Had heard of Guillain-Barre Syndrome before: (*n* = 218)	16 (33.3%)	32 (20.4%)	28 (24.1%)	4 (10.5%)
Think they have enough information about Zika: (*n* = 214)	24 (51.1%)	50 (32.3%)	36 (31.6%)	13 (34.2%)
Want more information about Zika: (*n* = 145)	21 (84.0%)	91 (82.7%)	68 (86.1%)	22 (75.9%)
^a^Would like more information about the: (*n* = 203)				
Causes of Zika	21 (44.7%)	93 (89.4%)	65 (61.3%)	27 (73.0%)
Signs and Symptoms of Zika	22 (46.8%)	99 (95.2%)	75 (70.8%)	24 (64.9%)
Zika Prevention	23 (48.9%)	99 (95.2%)	72 (67.9%)	26 (70.3%)
Zika Treatment	23 (48.9%)	103 (99.0%)	76 (71.7%)	27 (73.0%)
Consequences of having Zika	19 (40.4%)	90 (86.5%)	67 (63.2%)	23 (62.2%)

*n* = number of participants who responded to survey question; *m* = number who provided the response indicated; *n* = number of participants who responded to survey question; ^a^=Multiple responses were possible for this question; Persons in the Pregnant group indicated that they were pregnant at the time of survey completion

*Knowledge of Zika Transmission* is not significantly associated with travel history (beta = 4.16, *p* = 0.13) but is significantly associated with pregnancy (beta = 8.4, *p* = 0.01) [Table 3]. Among all participants, the average score for this domain was 56 [53, 58] [Table 4]. When adjusted for having heard about Zika before taking the survey, on average pregnant persons scored 6.21 points higher (beta = 6.21, *p* = 0.04) than non-pregnant persons. The majority of participants (88% (180/204)) were able to identify that Zika is preventable and that everyone, regardless of age or pregnancy status was able to acquire Zika virus (86% (186/217)). Very few (3% (6/219)) were able to accurately identify all of the common modes of transmission of Zika virus. With regards to the modes of transmission, 86% (187/219) identified transmission via mosquito bite, 59% (130/219) identified transmission via sexual intercourse and 35% (77/219) identified transmission of Zika from mother-to-child. Only 9% (20/219) indicated that they did not know how Zika is transmitted.

**Table 3:** Examining the association between Zika knowledge domains and participant or survey characteristics

		Knowledge of Zika transmission					Knowledge of Zika complications			
			Unadjusted		Adjusted^a^		Unadjusted		Adjusted^a^	
Model number	Demographic characteristics		β	*P*-value	β	*P*-value	β	*P*-value	β	*P*-value
1	Pregnancy		8.4	0.01*	6.21	0.04*	7.5	0.06	6	0.13
2	Travel History		4.16	0.13	2.49	0.34	−1.98	0.58	−3.26	0.36
3	Primary Language	Spanish	−7	0.01*	−6.28	0.01*	−3.63	0.31	−3.31	0.35
4		Other	−17.14	<0.0001*	−11.1	0.005*	−10.79	0.03*	−6.08	0.27
5	Survey Language		−6.43	0.04*	−8.52	0.004*	1.74	0.67	0.37	0.92
6	Language mismatch		−5.25	0.04*	−1.53	0.56	−6.94	0.04*	−4.58	0.2
7	Translator use		−12	0.01*	−3.71	0.45	−22.51	0.0002*	−18.81	0.005*

Reference groups: Model 1 = non-pregnant; Model 2 = did not travel; Model 3 = primary language English; Model 4 = survey language English; Model 5 = participant’s primary language matched survey language (language match); Model 6 = did not use translator to fill out the survey

^a^ Model adjusted for having heard about Zika before taking the survey

*=*p* < 0.05 for t-test of linear regression parameters

**Table 4:** Average Zika knowledge scores, stratified by pregnancy status

Total participants (*N* = 223)		Pregnant			Not Pregnant	
	n	Mean knowledge score	95% CI for mean	n	Mean of knowledge score	95% CI for mean score
Knowledge of Zika Transmission	50	62	[57, 68]	158	54	[51, 57]
*Where is the transmission of Zika actively occurring?*	50	49	[41, 58]	154	37	[33, 42]
*Who can get Zika?*	50	91	[84, 99]	156	86	[82, 91]
*How does a person get Zika?*	50	71	[66, 75]	157	63	[59, 67]
*What are the signs and symptoms of Zika?*	49	44	[34, 55]	156	35	[30, 41]
*Does everybody who gets Zika show symptoms?*	46	74	[61, 87]	147	6	[52, 0.68]
*Can you prevent Zika?*	46	85	[74, 96]	146	89	[84, 94]
*How can you prevent Zika?*	49	55	[46, 65]	153	5	[45, 54]
*Is there treatment for Zika?*	50	32	[19, 45]	159	16	[11, 22]
Knowledge of Zika Guidelines	44	86	[83, 89]	141	85	[83, 87]
*If you have traveled to a country with Zika virus and then develop weakness or numbness, what should you do?*	40	100	.	131	94	[90, 98]
*If a non-pregnant person thinks that they have Zika, what should they do?*	45	91	[82, 100]	146	97	[95, 100]
*If a pregnant person thinks that they have Zika, what should they do?*	45	1	.	145	97	[95, 100]
*If the male partner in a couple has traveled to a Zika endemic area and has been diagnosed with Zika or has (or had) symptoms, what steps should the couple take to reduce the risk of transmission?*	41	75	[65, 85]	136	74	[69, 80]
*If the male partner in a couple has traveled to a Zika endemic area, and does not develop symptoms, what should the couple do reduce the risk of transmission?*	42	74	[67, 80]	132	75	[72, 78]
*If you are pregnant and travel to a Zika endemic area, what steps should you take to reduce the risk of transmission?*	40	73	[66, 79]	131	74	[71, 76]
Knowledge of Zika Complications	49	55	[48, 62]	157	48	[44, 51]
*If a pregnant woman has Zika, what are the risks she faces?*	48	68	[59, 77]	154	74	[69, 78]
*What is the correct definition for microcephaly?*	49	69	[56, 83]	152	57	[49, 65]
*Do you think there is a link between Zika and Microcephaly?*	48	71	[57, 84]	144	58	[49, 66]
*If a pregnant woman has Zika, what are the risks for her fetus / baby?*	47	68	[58, 79]	154	64	[58, 70]
*What is Guillain-Barre Syndrome?*	48	27	[14, 40]	153	15	[9, 21]
*Do you think there is a link between Zika and Guillain-Barre syndrome?*	44	23	[10, 36]	144	13	[8, 19]

Most participants (63% (129/204)) were able to correctly identify that not everyone who acquires Zika is symptomatic. However, the average score for the question on symptoms of Zika virus was 38 [33, 43], and few participants (2% (4/217)) were able to identify all of the symptoms. With regards to Zika symptoms, most identified fever (55% (120/217) and headache 42% (92/217). However, less than one third identified rash (31% (67/217)) and joint pain (29% (63/217) respectively) and only 15% (32/217) were able to identify conjunctivitis as a symptom.

*Knowledge of Zika Transmission* is significantly correlated with *Knowledge of Zika Guidelines* (*r* = 0.17, *p* = 0.02) and with *Knowledge of Zika Complications* (*r* = 0.34, *p* < 0.0001). *Knowledge of Zika Guidelines* and *Knowledge of Zika Complications* are not significantly correlated (*r* = 0.13 *p* = 0.06).

*Knowledge of Zika Complications* is not significantly associated with travel history (beta = −1.98, *p* = 0.58), or with pregnancy (beta = 7.5, *p* = 0.06) [Table 4]. Nearly half (46% (83/214)) identified all if the correct risks for pregnant women; however, pregnant persons had a lower average score regarding the risks that a pregnant woman faces if she has Zika (68 [59, 77]) than did non-pregnant persons (74 [69, 78]). When asked of the risks that pregnant women who have Zika face, some participants ((41% (87/214) identified that she may be sick and even fewer (34% (73/214)) identified that she may be at risk for a miscarriage. Few (10% (21/214)) identified that she may be at risk from illegal and/or unsafe termination of pregnancy.

More than half of participants (61% (134/218)) indicated that they have heard of microcephaly before with nearly all (94% (124/132)), able to accurately identify the correct definition. Similarly, most participants (95% (116/122)) believed that there was an association between Zika virus and microcephaly. However, more pregnant persons than non-pregnant persons were able to correctly identify the definition of microcephaly (97% (35/36)) and that there is a link between Zika virus and microcephaly. 42% (89/212) of all participants identified a baby being born with microcephaly as a risk posed to babies born from Zika infected mothers and 28% (60/212) identified the risk of the baby not growing or developing normally in the womb. Even fewer participants (14% (29/212)) identified the risk of miscarriage. Almost half of the respondents, (47% (99/212)), correctly identified all of the potential risks. The majority of participants (77% (168/218)) reported they had not heard of GBS prior to taking this survey, however many (79% (37/47) were able to identify the accurate definition of GBS and believed that there was a link between Zika virus and GBS (66% (22/35)).

The average score for *Knowledge of Zika Guidelines* among all participants was 85 [84, 87], (range 36 to 10). This score was the highest of the three domains among all participants, with 10% of participants scoring between 36 and 73 and 30% of participants scoring between 93 and 100. Within *Knowledge of Zika Guidelines*, the questions with the highest average scores were “If a pregnant person thinks they have Zika, what should they do” (98, [95, 100]) and “If a non-pregnant person thinks they have Zika, what should they do?” (96, [93, 99]). One of the questions with the lowest average scores in this domain to what a couple should do to reduce the risk of transmission when a male partner has traveled to a Zika endemic area but does not show symptoms (74, [72, 77]). Nearly three quarters of participants (74% (136/184)) correctly indicated that condoms should be used during intercourse if the male partner in a couple travels to a Zika endemic region and develops symptoms. However, only 35% (64/184) indicated that the couple should also refrain from intercourse for at least 6 months, in keeping with current CDC recommendations, in this situation [13]. The second lowest score in this domain pertained to the steps a pregnant person who has traveled to a Zika endemic area should take to reduce risk of transmission (74, [71, 76]), but over half of participants (55% (101/185)) correctly indicated that all of the following precautions as per CDC guidelines: take precautions to prevent mosquito bites, use condoms or refrain from intercourse while pregnant, obtain testing for Zika virus at the first prenatal care visit and delay travel to areas with Zika virus during pregnancy [14].

### Attitudes

Most participants (80% (165/207)) believe that Zika virus was an important issue in their community, but fewer pregnant persons than non-pregnant persons felt this way. A quarter (25% (51/201)) of all participants and 36% (16/45) of pregnant participants thought that a person who had Zika virus and their family would face stigmatization. Similarly, 30.2% (63/197) of all and 46% (20/44) of pregnant participants indicated that if woman has a baby with microcephaly or other disability, she would be stigmatized because of the child. Although both pregnant and non-pregnant persons are most worried that Zika virus can cause a baby to have disabilities (92% (185/201)), a greater proportion of pregnant persons (96% (42/44)) than non-pregnant (90% (130/145)) persons indicated this concern [Table 5].

**Table 5:** Participant attitudes towards Zika virus, stratified by pregnancy status and gender

Total participants (*N* = 223)	Pregnant	Non-pregnant		
		All	Females	Males
	m (%)	m (%)	m (%)	m (%)
Think that Zika is an important issue/problem in their community: (*n* = 207)	33 (71.7%)	121 (90.7%)	89 (80.9%)	29 (78.4%)
Feels that if person gets Zika, they and their family are discriminated or stigmatized because of it: (*n* = 201)	16 (36.4%)	33 (22.6%)	23 (21.7%)	9 (24.3%)
Feels that if a woman has a baby that has microcephaly or another disability, she will be discriminated against or stigmatized because of the child: (*n* = 197)	20 (46.5%)	42 (29.4%)	31 (30.1%)	11 (28.9%)
^a^Participants are most worried that Zika: (*n* = 201)				
Zika can make you sick	18 (40.9%)	112 (77.2%)	80 (75.5%)	31 (86.1%)
Zika can kill you	16 (36.4%)	106 (73.1%)	74 (69.8%)	31 (86.15)
Zika can cause babies to have disabilities	42 (95.5%)	130 (89.7%)	93 (87.7%)	34 (94.4%)
Zika can cause adults to have disabilities	14 (31.8%)	90 (62.1%)	64 (60.4%)	25 (69.4%)
Zika can be sexually transmitted	21 (47.7%)	111 (76.6%)	80 (75.5%)	30 (83.3%)
Zika will cause my child to be sick	25 (56.8%)	91 (62.8%)	64 (60.4%)	26 (72.2%)
Safe abortion is not available to me if I get Zika when pregnant	10 (22.7%)	81 (55.9%)	54 (50.9%)	26 (72.2%)

*n* = number of participants who responded to survey question; *m* = number who provided the response indicated; *n* = number of participants who responded to survey question; ^a^=Multiple responses were possible for this question; Persons in the “Pregnant” group indicated that they were pregnant at the time of survey completion

### Practices

Over half of the participants (57% (118/208)), and the majority of pregnant participants (81% (48/39)) reported they have taken action to prevent themselves from getting Zika virus. The most common actions taken by participants were removing standing or stagnant water (28% (57/204)), putting screens on windows or doors (24% (49/204)), and wearing covering clothing (23.0% (47/204)). Participants reported more actions related to the reduction of potential mosquito breeding sites (94% (154/164)) and protection against mosquito-borne transmission (63% (104/164)) than of protection against sexual transmission (23% (38/164)). Few respondents (17% (28/164)) reported using a condom in all sexual relations. Pregnant persons reported more actions related to the protection against mosquito-borne transmission than did non-pregnant persons, while non-pregnant persons indicated taking more actions related to the reduction of potential mosquito breeding sites. Importantly, less than 20% of pregnant persons but nearly 60% of non-pregnant persons indicated taking the actions related to the protection against sexual transmission of Zika virus [Table 6].

**Table 6:** Zika related practices reported by study participants, stratified by pregnancy status and gender

Total participants (*N* = 223)	Pregnant	Non-pregnant		
		All	Females	Males
	m (%)	m (%)	m (%)	m (%)
Since you heard about Zika, have you taken any action to prevent yourself from getting Zika? (*n* = 208)	38 (80.9%)	73 (49.0%)	58 (53.7%)	13 (34.2.%)
^a^What action have you taken to prevent yourself/your household from getting Zika? (*n* = 204)				
Reduction of potential mosquito breeding sites	45 (121.6%)	140 (164.7%)	114 (175.4%)	24 (133.3%)
Protection against mosquito-borne transmission	39 (105.4%)	83 (97.6%)	67 (103.1%)	15 (83.3%)
Protection against sexual transmission	7 (18.9%)	37 (43.5%)	26 (40.0%)	10 (55.6%)
Other	19 (51.4%)	73 (85.9%)	60 (92.3%)	13 (72.2%)
None of the above	8 (21.6%)	7 (8.2%)	4 (6.2%)	3 (16.7%)
^a^If you travel to a Zika endemic country, what action have you taken to prevent yourself/your household while abroad? (*n* = 164)				
Reduction of potential mosquito breeding sites	50 (172.4%)	190 (154.5%)	139 (156.2%)	47 (261.1%)
Protection against mosquito-borne transmission	51 (175.9%)	164 (133.3%)	123 (138.2%)	40 (222.2%)
Protection against sexual transmission	21 (72.4%)	73 (59.3%)	44 (49.4%)	29 (161.1%)
Other	19 (65.5%)	102 (82.9%)	78 (87.6%)	22 (122.2%)
None of the above	4 (13.8%)	10 (8.1%)	13 (14.6%)	6 (33.3%)

*n* = number of participants who responded to survey question; *m* = number who provided the response indicated; *n* = number of participants who responded to survey question; ^a^=Multiple responses were possible for this question; Reduction of potential mosquito transmission includes: Cleaned/scrubbed or covered water source/storage unit/water container(s), Removed standing water / stagnant water, Sprayed or fumigated my home, Cleaned household environment. Protection against mosquito-borne transmission includes used a mosquito net, worn covering clothing, put screens on windows or doors. Protection against sexual transmission includes: used a condom in all sexual relations, abstained from sexual intercourse. Other includes: Drank clean water, washed in clean water, prayed to God; Persons in the Pregnant group indicated that they were pregnant at the time of survey completion

## Discussion

Developing an understanding of a community’s knowledge of Zika virus can be an important tool in forming future Zika interventions and educational materials. This survey helped in elucidating the knowledge of and attitudes towards Zika held by NYC community members and provides insight on the association between knowledge, attitudes and practices and pregnancy status and travel history. The results from our study indicate pregnancy is positively associated with *Knowledge of Zika Transmission*, as hypothesized, but not other knowledge scores, and travel status is not significantly associated with Zika knowledge.

Importantly, our results suggest that while the majority of participants heard of Zika prior to this survey, there are gaps in knowledge and information regarding multiple aspects of Zika virus including transmission risks, prevention methods, and symptoms in our sample population. Most participants were aware that Zika virus can be transmitted via mosquito bites but other forms of transmission were less well known. Although our study reveals that people believe that Zika virus is an important issue in their community, only about 60% of people think that it is possible to get Zika in their community, suggesting that 40% of persons do not think that the risk of Zika applies to them. However, persons living in NYC can still become infected with Zika when traveling abroad or through sexual transmission of travel-associated Zika. Overall, about 40% or participants did not know that Zika can be sexually transmitted, but 72% of people were concerned about sexual transmission of Zika. This may reflect fear about the possibility of sexual transmission in the absence of personally knowing that Zika can be sexually transmitted. Of the persons who think that Zika is an issue in their community 37% of did not know that Zika was sexually transmitted, and 42% of persons who did not think that Zika was an issue in their community did know that Zika was sexually transmitted. While there is an overall concern about Zika within this group, these results suggest that some people may not be protecting themselves from sexually transmitted Zika. This finding is consistent with participant reported practices; 20% of participants who both thought that Zika was an issue in their community and knew about sexual transmission of Zika did not take any actions to protect themselves against Zika. In addition, larger knowledge gaps existed with regards to symptoms of Zika virus infection. Though most participants were able to identify some of the symptoms of Zika virus infection, the overwhelming majority were not able to identify all of them. Participants indicated that the aspect of Zika that was most concerning to them was its risk of congenital birth defects. Despite this concern, a smaller percentage of individuals were able to identify mother-to-child transmission as a risk factor. This information indicates the importance in promoting Zika virus education around prenatal care. Pregnant persons were more familiar with both microcephaly and Guillain-Barré syndrome than were non-pregnant persons, signaling that future public health interventions should clearly communicate that, despite the serious pregnancy complications, Zika poses risks for pregnant and non-pregnant persons alike.

The results from our study also demonstrate that some individuals associate Zika virus with stigma to those who are infected as well as those who have infants with congenital birth defects. This stigma may generate fear that could prevent individuals who feel they have been exposed to or have acquired Zika virus from seeking appropriate medical care. Educational interventions should be careful to address and help diminish stigmatization. Large knowledge gaps also existed around ways to prevent Zika virus transmission. A little over half of our participants indicated that they have taken precautions to prevent themselves from Zika virus transmission since hearing about the virus. Particularly striking was the difference reported between pregnant females and non-pregnant males; approximately 80% of pregnant females compared to only about 34% of non-pregnant males reported taking action to protect themselves from Zika. Because of the potential for sexual transmission and the development of Guillain-Barré syndrome, future public health interventions should stress the importance of protection against Zika for all people, not only pregnant individuals. Despite the fact that over half of all participants were able to identify that Zika virus is sexually transmitted, only a small percentage of the participants identified condom use as a precaution one should take if one is pregnant and traveling to a Zika endemic region. More participants identified condom use as a method to prevent Zika virus, but when asked what they would do in specific scenarios to prevent Zika transmission, condom use was not as commonly evoked. This discrepancy in behaviors and attitudes surrounding sexual and reproductive health in our participants perhaps illustrates a gap in knowledge and practice regarding Zika virus prevention; specifically, that condom use, an underused method to prevent transmission, can directly prevent what participants find most concerning about Zika—the birth of children with congenital anomalies.

An unexpected result was that knowledge scores significantly differed based on the primary language of the survey respondent. Linear regression showed that participant primary language, the survey language, a mismatch between participant’s primary language and the language they took the survey in, and use of a translator were negatively associated with Zika knowledge. If reflective of a real difference, future educational materials should be made available in many languages. It is possible that if not reflective of a real-life difference in knowledge, the negative association between language factors and knowledge could have been a methodological artifact. For example, although the survey was forward and back translated by a native Spanish speaker, there could be a problem of the translation of some of the questions. Additionally, the strong negative association between knowledge and translator use could be because a result of the fact that translators was not professional translators but were a family member or friend who helped translate. Future KAP surveys should assess if non-native English speakers truly have less Zika knowledge than English speakers, and if so, educational interventions should be accessible to non-English speakers.

Our study informs public health efforts, however there are limitations to consider. Due to the importance of rapidly obtaining information, administration of our survey was limited to a few areas in NYC, concentrated around Northern Manhattan and the Bronx, and was a small sample size. Although distribution to participants in multiple different study locations allowed for a wider sampling of the Northern Manhattan and Bronx population, it could have resulted in an unmeasured correlated data structure. Unfortunately, the location at which the survey was filled out was not recorded, and thus potential clustering or correlation of errors by study site was not accounted for in this analysis. Convenience sampling was used, which may have created bias. Some of the participants recruited were in a hospital setting, which may lack representation of the community in general and additional bias as these women are potentially more informed about Zika virus and its complications.

Because of the number of missing responses, each question was analyzed using different samples, and we must consider that the associations seen are due to the different samples (different people) or due to a real association. Another limitation is that only some of the survey questions included “I don’t know” as a possible answer response. This limited our ability to determine if the question was missing because people did not know the answers or skipped the question for another reason (such as thinking they knew the right answer but did not see their response listed as an option). This may have contributed to the number of missing responses.

Additionally, because of the ongoing outbreak, information such as areas of transmission as well as recommendations for prevention were rapidly developing and changing, making it difficult to accurately assess knowledge and practices of participants. Though explained to the participants in written and verbal instructions, there may have been misinterpretation in the instructions of the survey as several questions included multiple responses, while others included one appropriate response. These effects might be mitigated by the short four-month time frame during which the survey was administered, which may reduce the effects that media exposure and guideline updates would have on survey results. Despite these limitations, our study was able to capture information of a sample of NYC habitants, including those traveling to endemic regions and pregnant women.

### Public health implications

This survey provides both direct information about what participants want to know and where there are gaps in the community’s Zika knowledge. Our results demonstrate the importance of educational efforts particularly with respect to sexual and reproductive health to serve the concerns people have regarding congenital abnormalities associated with Zika virus as well as the lack of knowledge surrounding prevention methods. Our results also highlight the fact that a majority of community members want more information on Zika virus, indicating a desire for more knowledge on causes, symptoms, prevention and consequences. These aspects correspond with the lower knowledge scores, suggesting that targeted efforts to increase knowledge should be undertaken. Being that only a minority of participants indicated that they first heard of Zika virus from “healthcare worker/ private doctor or pharmacy,” suggests that increased educational endeavors led by local healthcare providers are needed but also suggests that media outlets are an important source of Zika information and educational content should be bolstered. The development of sustainable public health strategies aimed at bridging these gaps in knowledge, reducing the stigmatization surrounding Zika virus, and improving practices in treatment and prevention, warrant further investment.

## Conclusion

The lack of knowledge surrounding Zika as well as insufficient practices in Zika prevention indicate the necessity for sustained public health educational efforts in Zika virus. Programs focused concerns of sexual health and maternal-fetal health with respect to Zika as well as those that are able to penetrate stigmatization and other cultural barriers will be particularly useful in addressing the existing desire for more information. Additionally, further studies regarding community knowledge, attitudes and practices around Zika virus in a variety of locations, especially in among groups that live in or frequently travel to Zika endemic regions worldwide, will be especially useful in addressing this growing global health concern.

## Additional file

Additional file 1: Elaborated Statistical Analysis Methods. Provides detailed information regarding the organization of questions into domains and the statistical methods used in analysis. (DOCX 39 kb)

## Abbreviations

CDC: Centers for Disease Control and Prevention
GBS: Guillain-Barré Syndrome
KAP: Knowledge, Attitudes, and Practices
NYC: New York City
PAHO: Pan American Health Organization
WHO: World Health Organization
