# Supplementary material for: A survey of the knowledge, attitudes and practices on Zika virus in new York City
Source: BMC Public Health. 2018 Jan 2;18:98. doi: 10.1186/s12889-017-4991-3 (PMC5748954; doi:10.1186/s12889-017-4991-3)
Supplement: Additional file 1: — Elaborated Statistical Analysis Methods. Provides detailed information regarding the organization of questions into domains and the statistical methods used in analysis. (DOCX 39 kb) [file 12889_2017_4991_MOESM1_ESM.docx]

# Appendix I: Elaborated Statistical Analysis Methods

*Domains:*

*Participant Characteristics:* Questions in this category determined participant age, gender, primary language, New York City borough of residence, and participant birthplace. These questions also assess if participants traveled to the Caribbean, Central or South America within the last year (i.e. during the Zika epidemic in this region), and if participants were pregnant at the time of taking the survey.

*Knowledge:* The domain *General Zika Knowledge* primarily focuses on determining if participants had heard about Zika, Microcephaly or Guillain-Barre syndrome before taking the survey. It also includes questions to determine when and where participants first heard about Zika, if they wanted more information about Zika, and if so, what they wanted more information about. *Knowledge of Zika Transmission* assesses participant knowledge of Zika transmission, symptoms, prevention and treatment. It includes questions asking where Zika transmission is occurring, who can get Zika, how persons get Zika and if and how it can be prevented, and questions about symptomatology and treatment. All questions in this domain can be found in Table 4. The *Knowledge of Zika Guidelines* domain asks participants to indicate what should be done in hypothetical situations related to risk of Zika infection. For example, one question asks what a pregnant person who has traveled to a Zika endemic area should do to reduce transmission risk. All of the questions in this domain can be found in Table 4 in the manuscript. Correct answers to these hypotheticals reflected the CDC and WHO guidelines for Zika prevention and response at time of survey implementation. The domain *Knowledge of Zika Complications* assessed participant knowledge of Zika associated complications. It includes questions regarding the risks faced by a pregnant person and the fetus/baby if infected with Zika. It also includes questions assessing participant knowledge of Microcephaly and Guillain-Barré syndrome, and their association with Zika infection.

*Attitudes:* Questions in this category assessed participant opinions, feelings, and concerns regarding Zika and Zika related stigma, and can be found in Table 5 in the manuscript.

*Practices:* Questions in this category attempted to determine the preventative actions utilized by participants since hearing about Zika, both at home and while traveling and can be found in Table 6 in the manuscript.

*Question Structure*

Within this survey there were 5 main types of multiple-choice question structures; Yes/No, Select One. Multiple Component, All Of The Above, and Conditional. Regardless of question structure, only some questions offered “I don’t know” as an answer choice. [Table 7].

| **Table 7: Survey Question Structures** | |
| --- | --- |
| **Question structure type** | **Description** |
| Yes/No | Either yes or no is the correct answer |
| Select One | There is only one correct answer choice |
| Multiple Component | A specific combination of multiple answer choices was correct |
| All Of The Above | All of the given answer choices were correct; there was an option to select “All of the above” |
| Conditional | Answer the following question only if provided a specific answer to a preceding question (branching question) |

The way in which the responses to the individual questions within the scored knowledge domains varied depending on the response style and the presence of an “I don’t know” response option. Possible scores for the individual questions ranged from 0 to 1. For Yes/No or Select One questions, participants received a score of 1 if they selected the correct answer and a score of 0 for selecting the incorrect answer. For Multiple Component Questions, participants received 1 point for circling a correct repose choice, 1 point for not circling an incorrect response choice, and 0 points for circling an incorrect response choice. The score for each individual question within the domain was calculated as the average of the correct answer choices for that question [Table 8].

| **Table 8: Comparison of Knowledge Domain Score Scoring Systems** | | | | | | | | | |
| --- | --- | --- | --- | --- | --- | --- | --- | --- | --- |
| **Knowledge of Zika Transmission** | **N Miss** | **N** | **Mean** | **95% CI for Mean** | | **Std Dev** | **Variance** | **Min** | **Max** |
| Listwise deletion | 33 | 191 | 0.58 | [0.55, 0.60] | | 0.17 | 0.03 | 0.13 | 0.94 |
| At least 2 non-missing | 3 | 221 | 0.56 | [0.53, 0.58] | | 0.19 | 0.04 | 0.00 | 0.94 |
| At least half non-missing | 3 | 221 | 0.56 | [0.53, 0.58] | | 0.19 | 0.04 | 0.00 | 0.94 |
|  |  |  |  |  |  |  |  |  |  |
| **Knowledge of Zika Guidelines** | **N Miss** | **N** | **Mean** | **95% CI for Mean** | | **Std Dev** | **Variance** | **Min** | **Max** |
| Listwise deletion | 74 | 150 | 0.86 | [0.84, 0.87] | | 0.10 | 0.01 | 0.41 | 1.00 |
| At least 2 non-missing | 16 | 208 | 0.86 | [0.84, 0.87] | | 0.11 | 0.01 | 0.36 | 1.00 |
| At least half non-missing | 28 | 196 | 0.85 | [0.84, 0.87] | | 0.11 | 0.01 | 0.36 | 1.00 |
|  |  |  |  |  |  |  |  |  |  |
| **Knowledge of Zika Complications** | **N Miss** | **N** | **Mean** | **95% CI for Mean** | | **Std Dev** | **Variance** | **Min** | **Max** |
| Listwise deletion | 48 | 176 | 0.47 | [0.44, 0.51] | | 0.23 | 0.05 | 0.07 | 1.00 |
| At least 2 non-missing | 5 | 219 | 0.49 | [0.46, 0.52] | | 0.25 | 0.06 | 0.00 | 1.00 |
| At least half non-missing | 5 | 219 | 0.49 | [0.46, 0.52] | | 0.25 | 0.06 | 0.00 | 1.00 |
| **Scores were compiled using 3 different systems to see if there was an effect on the mean scores based on the way the scores were compiled. The system for at least half non-missing was selected as the scoring system.** | | | | | | | | | |

*Missing Variables*

Questions were considered missing if respondents did not circle any answer choices. For questions containing an “I don’t know” response option, participants received a score of 0 for the question if “I don’t know” was their only response, but were scored in the standard manner if they included other response. Participants received 0 for conditional questions if their answer to the parent question resulted in instruction to skip the question. For example, if participants indicated that they had never heard of Microcephaly before, they were instructed to skip, and received 0 points for, the following questions about the definition of microcephaly and its link with Zika virus.

In order to determine if the number of missing responses affected the overall average knowledge score for each of the domains, knowledge scores were compiled using three different scoring techniques and the means were under each system were compared. In all techniques, the knowledge score was calculated as the average of the non-missing scores on the individual question scored, but the denominator of this average differed depending on the scoring system. Using listwise deletion persons missing any of the questions received a missing domain score. While the scores in the at least half non-missing was calculated as the average score of the questions for persons with at least half and at least 2 non-missing questions. The distributions of the continuous scores under each method can be seen in Table 8. Because the means were similar, the method utilizing the scores for at least half non-missing was selected for analysis [Tables 9, 10, and 11].

| Table 9: Demographic Survey Questions | | | | | | | | | |
| --- | --- | --- | --- | --- | --- | --- | --- | --- | --- |
| English | | | | **Español** | | | | | |
| Number Missing | **Proportion Missing (%)** |  |  | **Number Missing** | Proportion Missing (%) | |  | |  |
| Demographic Information | | | | **Información General** | | | | | |
| 15 | 8 | Date | | 10 | 21 | Fecha | | |  |
| 0 | 0 | What is your primary language? | | 0 | 0 | Cual es su lenguaje primario? | | | |
| 3 | 2 | Are you using a translator to help you fill out this survey? | | 1 | 2 | Estas usando un interprete para ayudarte a completar este cuestionario? | | | |
| 1 | 1 | Gender | | 2 | 4 | Genero | | | |
| 6 | 3 | Age | | 10 | 21 | Edad | | | |
|  |  | Birthplace (country) | |  |  | Lugar de nacimiento (pais) | | | |
|  |  | Current City | |  |  | Ciudad Actual | | | |
| 6 | 3 | If you reside in New York City, which borough/region do you live in? | | 2 | 4 | Si reside en la ciudad de Nueva York, en que Municipio/Region vive usted? | | | |
| 0 | 0 | Have you traveled to the Caribbean, Central America or South America within the last year? | | 0 | 0 | Ha viajado usted al Caribe, America Central, o Sur America en el ultimo año? | | | |
|  |  |  | Which country? |  |  |  | | Cual pais? | |
| 3 | 2 |  | When was the last time you traveled to the Caribbean, central America or South America? | 0 | 0 |  | | Cuando fue la ultima vez que usted viajo al Caribe, America Central, o Sur America? | |
| 9 | 5 | Are you currently pregnant or may be pregnant? | | 5 | 11 | Esta embarazada actualmente o podrias estar embarazada? | | | |
| 13 | 7 |  | How far along is your pregnancy? | 5 | 11 |  | | | Cuan avanzado esta su embarazo? |

| Table 10: Attitude and Practices Questions | | | | | | | | | | |
| --- | --- | --- | --- | --- | --- | --- | --- | --- | --- | --- |
| English | | | | | **Español** | | | | | |
| Number Missing | **Proportion Missing (%)** | |  |  | **Number Missing** | **Proportion Missing (%)** | | |  |  |
| Attitudes | | | | | Actitudes | | | | | |
| 13 | 7 | Do you think that Zika is an important issue / problem in your community? | | | 4 | 9 | ¿Piensa usted que el Zika es asunto importante/ | | | |
| 18 | 10 | If a person gets Zika, are they and their family discriminated or stigmatized because of it? | | | 5 | 11 | Si a una persona le da el Zika, ¿Estan ellos y sus familiares discriminados o señalados por esto? | | | |
| 20 | 11 | If a woman has a baby that has microcephaly or another disability, is she discriminated against or stigmatized because of the child? | | | 7 | 15 | Si una mujer tiene un bebe con microcefalia u otra incapacidad, ¿Se discrimina contra ella o es señalada por el bebe? | | | |
| 19 | 11 | What worries or concerns you most about Zika? | | | 4 | 9 | Que es lo mas que le preocupa sobre el Zika? | | | |
| Practices | | | | | Practicas | | | | | |
| 14 | 8 | Since you heard about Zika, have you taken any action to prevent yourself from getting Zika? | | | 2 | 4 | | Desde que escucho del Zika, ha tomado usted alguna accion para prevenir que contraiga el Zika? | | |
| 16 | 9 | What action have you taken to prevent yourself / your household from getting Zika while in US? | | | 4 | 9 | | ¿Que accion ha tomado usted/su hogar para prevenir tener Zika mientras estas/estan en Estados Unidos? | | |
| 55 | 31 | If you travel to a Zika endemic country what action have you taken to prevent yourself / your household from getting Zika in your household while abroad? | | | 5 | 11 | | Si viaja a un pais endemico con Zika, que accion ha tomado usted y su familia para prevenir contraer Zika durante su estadia en el estranjero? | | |

| Table 11: Scored Knowledge Survey Questions | | | | | | | | | | | | | | |
| --- | --- | --- | --- | --- | --- | --- | --- | --- | --- | --- | --- | --- | --- | --- |
| English | | | | | | | | Español | | | | | | |
| Number Missing | **Proportion Missing (%)** | | | |  | |  | Number Missing | Proportion Missing (%) | | |  | |  |
| General Zika Knowledge | | | | | | | | | | | | | | |
|  |  | | | Have you heard of Zika before? | | | |  |  | Habias escuchado del Zika anteriormente? | | | | |
| 4 | 2 | | |  | | | When did you first hear about Zika? | 1 | 2 |  | | | Cuando fue la primera vez que escuchastes del Zika? | |
| 3 | 2 | | |  | | | Where/from whom did you first hear about Zika? | 1 | 2 |  | | | Donde / De quien escucho usted sobre el Zika? | |
| 7 | 4 | | | Do you think it is possible to get Zika in your community/local area now? | | | | 6 | 13 | Crees que es posible obtener Zika en tu comunidad/ area local ahora? | | | | |
| 4 | 2 | | | Have you heard of "Microcephaly" before? | | | | 2 | 4 | Has escuchado de Microcefalia antes? | | | | |
| 4 | 2 | | | Have you heard of Guillain-Barré Syndrome before? | | | | 2 | 4 | Has escuchado del Sindrome de Guillain-Barre anteriormente? | | | | |
| 8 | 5 | | | Do you think you have enough information about Zika? | | | | 2 | 4 | ¿Piensa usted que tiene suficiente informacion sobre el Zika? | | | | |
| 13 | 7 | | | |  | Do you want more information about Zika? | | 5 | 11 |  | | ¿Quiere mas informacion sobre el Zika? | | |
| 16 | 9 | | | |  | What would you like more information about? | | 5 | 11 |  | | ¿De que mas le gustaria tener informacion? | | |
| Knowledge of Zika Transmission | | | | | | | | | | | | | | |
| 6 | 3 | | Where is the transmission of Zika Virus actively occurring? | | | | | 2 | 4 | Donde esta ocurriendo activamente la transmission del Zika? | | | | |
| 4 | 2 | | Who can get Zika? | | | | | 1 | 2 | A quien le puede dar el Zika? | | | | |
| 3 | 2 | | How does a person get Zika? | | | | | 1 | 2 | Como a una persona le da Zika? | | | | |
| 6 | 3 | | What are the signs and symptoms of Zika? | | | | | 1 | 2 | Cuales son las señales y sintomas del Zika? | | | | |
| 14 | 8 | | Does everybody who gets Zika show symptoms? | | | | | 6 | 13 | A todo el que le da Zika, muestra sintomas? | | | | |
| 15 | 8 | | Can you prevent Zika virus? | | | | | 5 | 11 | Puedes prevenir el virus del Zika? | | | | |
| 8 | 5 | | | |  | | How can you prevent Zika? | 2 | 4 |  | | | Como puedes prevenir el Zika? | |
| 7 | 4 | Is there treatment for Zika? | | | | | | 4 | 9 | Hay tratamiento para el Zika? | | | | |
| Knowledge of Zika Guidelines | | | | | | | | | | | | | | |
| 38 | 21 | If you have traveled to a country with Zika virus and then develop weakness or numbness, what should you do? | | | | | | 7 | 15 | | Si viajas a un pais con el virus de Zika y desarrollas debilidad y adormecimiento, ¿Que debes hacer? | | | |
| 17 | 10 | If a non-pregnant person thinks that they have Zika, what should they do? | | | | | | 6 | 13 | | Si una persona no embarazada piensa que tiene Zika, ¿Que debe hacer? | | | |
| 16 | 9 | If a pregnant person thinks that they have Zika, what should they do? | | | | | | 7 | 15 | | Si una persona embarazada piensa que tiene Zika, Que debe hacer? | | | |
| 33 | 19 | If you are pregnant and travel to a Zika endemic area, what steps should you take to reduce the risk of transmission? | | | | | | 6 | 13 | | Si estas embarazada y viajas a un pais endemico con Zika, que pasos debes tomar para reducir el riesgo de transmision? | | | |
| 31 | 18 | If the male partner in a couple has traveled to a Zika endemic area and has been diagnoses with Zika or has (or had) symptoms, what steps should the couple take to reduce the risk of transmission? | | | | | | 8 | 17 | | Si el compañero masculino en una pareja ha viajado a un area endemic con Zika y ha sido diagnosticado con Zika o tiene o ha tenido sintomas, ¿Que pasos debe tomar la pareja para reducir los riesgos de transmission? | | | |
| 35 | 20 | If the male partner in a couple has traveled to a Zika endemic area, and does not develop symptoms, what should the couple do to reduce the risk of transmission? | | | | | | 8 | 17 | | Si el compañero masculino en una pareja ha viajado a un area endemica con Zika, y no desarrolla sintomas, Que debe hacer la pareja para reducer los riesgos de transmission? | | | |
| Knowledge of Zika Complications | | | | | | | | | | | | | | |
| 7 | 4 | | If a pregnant woman has Zika, what are the risks she faces? | | | | | 3 | 6 | | Si una mujer embarazada tiene Zika, cuales son los riesgos que ella enfrenta? | | | |
| 7 | 4 | | What is the correct definition for microcephaly? | | | | | 3 | 6 | | Cual es la definicion correcta de microcefalia? | | | |
| 16 | 9 | | Do you think there is a link between Zika and Microcephaly? | | | | | 7 | 15 | | Cree usted que hay una coneccion entre Zika y Microcefalia? | | | |
| 8 | 5 | | If a pregnant woman has Zika, what are the risks for her fetus / baby? | | | | | 4 | 9 | | Si una mujer embarada tiene Zika, cuales son los riesgos para el feto / bebe? | | | |
| 7 | 4 | | What is Guillain-Barre Syndrome? | | | | | 3 | 6 | | ¿Que es el Sindrome de Guillain-Barre? | | | |
| 21 | 12 | | Do you think there is a link between Zika and Guillain-Barre syndrome? | | | | | 3 | 6 | | ¿Crees que hay una coneccion entre Zika y el Sindrome de Guillain-Barre? | | | |
